# Surface properties of 1T-TaS_2_ and contrasting its electron-phonon coupling with TlBiTe_2_ from helium atom scattering

**DOI:** 10.3389/fchem.2023.1249290

**Published:** 2023-11-16

**Authors:** Philipp Maier, Noah. J. Hourigan, Adrian Ruckhofer, Martin Bremholm, Anton Tamtögl

**Affiliations:** ^1^ Institute of Experimental Physics, Graz University of Technology, Graz, Austria; ^2^ Department of Chemistry and iNANO, Aarhus University, Aarhus, Denmark

**Keywords:** transition metal dichalcogenide, topological insulator, charge density wave, helium atom scattering, electron-phonon coupling, thermal expansion

## Abstract

We present a detailed helium atom scattering study of the charge-density wave (CDW) system and transition metal dichalcogenide 1T-TaS_2_. In terms of energy dissipation, we determine the electron-phonon (e-ph) coupling, a quantity that is at the heart of conventional superconductivity and may even “drive” phase transitions such as CDWs. The e-ph coupling of TaS_2_ in the commensurate CDW phase (*λ* = 0.59 ± 0.12) is compared with measurements of the topo-logical insulator TlBiTe_2_ (*λ* = 0.09 ± 0.01). Furthermore, by means of elastic He diffraction and resonance/interference effects in He scattering, the thermal expansion of the surface lattice, the surface step height, and the three-dimensional atom-surface interaction potential are determined including the electronic corrugation of 1T-TaS_2_. The linear thermal expansion coefficient is similar to that of other transition-metal dichalcogenides. The He−TaS_2_ interaction is best described by a corrugated Morse potential with a relatively large well depth and supports a large number of bound states, comparable to the surface of Bi_2_Se_3_, and the surface electronic corrugation of 1T-TaS_2_ is similar to the ones found for semimetal surfaces.

## 1 Introduction

While it has long been known that the weak short-range van der Waals (vdW) interaction holds together layered materials like graphite and MoS_2_ (Fiedler et al., 2023), with the availability of graphene by the so-called scotch tape technique, a vast class of two-dimensional (2D) materials has been investigated (Duong et al., 2017). Mechanical cleavage can be used to prepare monolayers of vdW layered materials, a preparation technique that also works for the layered class of 3D topological insulators (TIs), and first attempts to use these materials in vdW heterostructures and devices are described in Liu et al. (2016). The well-known physics and chemistry of three-dimensional bulk matter often become irrelevant for 2D materials, revealing exotic phenomena in vdW-layered crystals (Duong et al., 2017). Among the most prominent 2D materials are the semimetal graphene, and the transition metal dichalcogenides (TMdCs), which tend to be semiconductors. Here, we provide a detailed helium atom scattering (HAS) study of the TMdC and archetypal charge-density wave (CDW) system 1T-TaS_2_ (Rossnagel, 2011). The electron-phonon coupling of the latter is further compared with measurements of the topological insulator TlBiTe_2_, which in contrast exhibits a stronger bonding between the layers. Scattering and diffraction from both surfaces are compared in terms of interlayer bonding and surface quality, and completed with measurements of the thermal expansion and a determination of the three-dimensional atom-surface interaction potential of 1T-TaS_2_.

**FIGURE 1:**
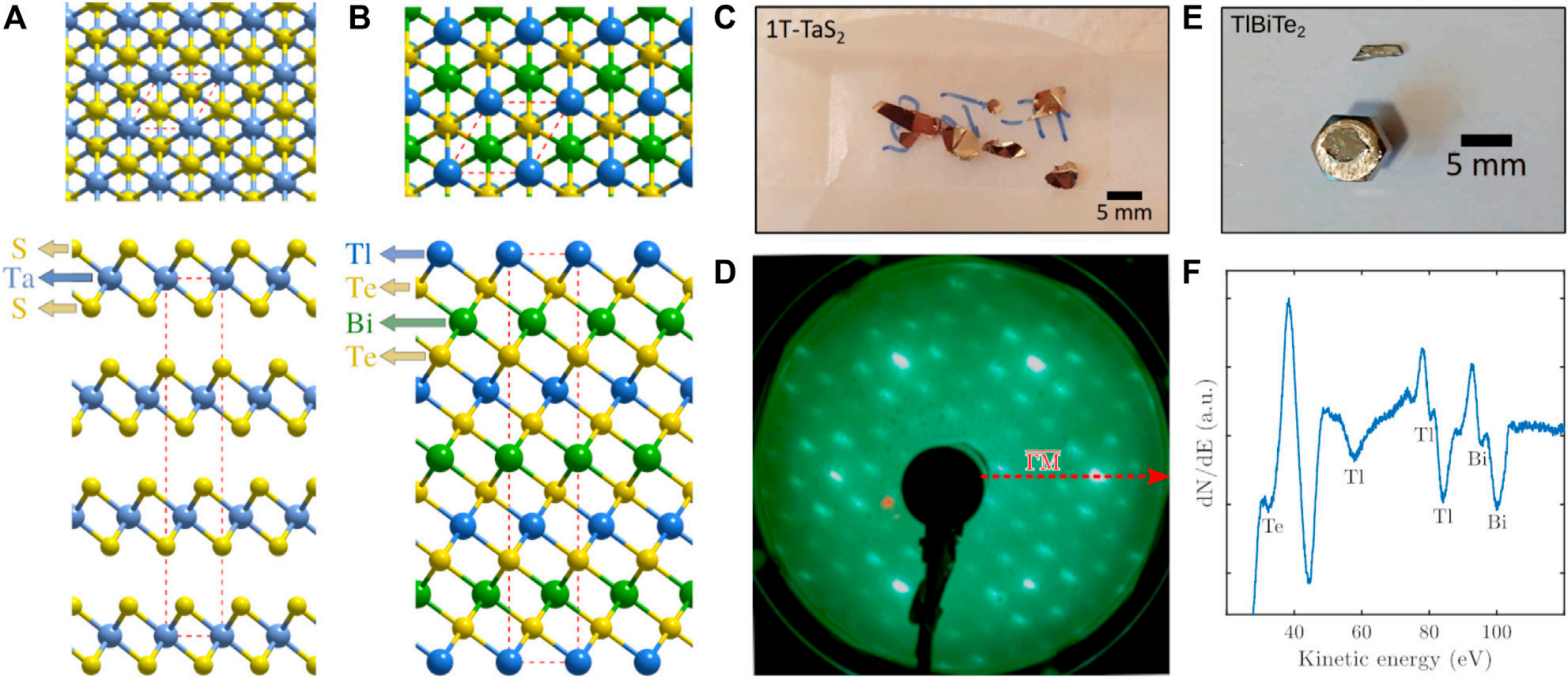
Top and side view of **(A)** 1T-TaS_2_(0001) and TlBiTe_2_(111) **(B)**. **(C)** Image of the as-grown 1T-TaS_2_ crystals. **(D)** shows the low energy electron diffraction (LEED) pattern of 1T-TaS_2_ with the sample aligned along the high symmetry $\bar{\Gamma M}$ orientation at room temperature. The six main (bright) spots according to the hexagonal crystal structure are surrounded by the so-called “star of David” pattern from corresponding clusters in the nearly commensurate charge-density wave phase. **(E)** Image of a TlBiTe_2_ crystal together with the cleavage post and the attached remaining crystal. **(F)** shows an Auger electron spectrum of the same crystal with signatures of mostly Tl and Bi.

TMdCs are atomically thin semiconductors of the type MCh_2_, with M being a transition metal atom and Ch a chalcogen atom, where one layer of M atoms is sandwiched between two layers of Ch atoms [see Figure 1A (Duong et al., 2017)]. A TMdC with a particular rich phase diagram is TaS_2_. The phase diagram of the 1T polytype of TaS_2_ (Figure 1A) involves several CDW transitions driven by strong electronic correlations and electron-phonon (e-ph) coupling upon changes of the surface temperature (Tsen et al., 2015; Vaskivskyi et al., 2015). No final agreement on the electronic ground state of the material or the role of correlations has been reached, and it has even been suggested that the existing experimental evidence for 1T-TaS_2_ is consistent with the ground state being a quantum spin liquid (Klanjšek et al., 2017; Law and Lee, 2017).

TlBiTe_2_ on the other hand is a trigonal crystal with 4 layers as shown in Figure 1B forming a repeated elemental sequence (Tl-Te-Bi-Te-Tl) and TlBiTe_2_ as well TlBiSe_2_ are thallium-based ternary narrow-band semiconductors (Singh et al., 2016). It was predicted theoretically that this class of materials are 3D TIs (Eremeev et al., 2010; Eremeev et al., 2011; Lin et al., 2010; Yan et al., 2010), later followed by experimental evidence of its topological properties (Chen et al., 2010; Kuroda et al., 2010; Kuroda et al., 2013). Chen et al. measured the 3D band structure of TlBiTe_2_ using angle-resolved photoemission spectroscopy (ARPES) and found a single Dirac cone in the center of the surface Brillouin zone (Chen et al., 2010; Singh et al., 2012). Due to the observation of the negative bulk band gap of −10 meV they concluded that TlBiTe_2_ may be classified as a semimetal rather than a narrow-gap semiconductor as suggested by theory and that this semimetallic nature explains its small thermoelectric power (Spitzer and Sykes, 1966; Kurosaki et al., 2003; Chen et al., 2010). Furthermore, according to Singh et al., Weyl semimetals can be realized at the topological critical point in alloys TlBi(S_1−*x*_Te_*x*_)_2_ by breaking the inversion symmetry in layer-by-layer growth in the order Tl-Te-Bi-S (Singh et al., 2012). However, such kind of structures have not been realized so far.

Similar to 1T-TaS_2_ and the 3D TI family Bi_2_Te_3_/Bi_2_Se_3_, where the quintuple layers are weakly bound by vdW interactions (Zhang et al., 2009), TlBiTe_2_ is also a layered material but its atomic layers are bonded covalently (Yan et al., 2010). While the e-ph coupling has been measured for a variety of TIs in particular the class of binary TIs (Ruckhofer et al., 2020), much less experimental information about the TlBiTe_2_ system is available not least in terms of the e-ph coupling.

## 2 Methods

HAS is ideally suited to study CDW phases since the neutral He beam is directly scattered by the surface electrons and HAS permits determination of the e-ph coupling *λ* (Manson et al., 2022) which specifies also conventional superconductivity (Benedek et al., 2020b). Previous HAS diffraction and phase transition data from HAS are described in Benedek et al. (2020b), including CDW systems with HAS (Tamtögl et al., 2019; Ruckhofer et al., 2023).

Our experiments were performed at the HAS apparatus in Graz. A detailed description of the experimental setup has been given elsewhere (Tamtögl et al., 2010). A nearly monochromatic beam (Δ*E*/*E* ≈ 2%) of ^4^He which is generated by a supersonic expansion through a cooled 10 μm nozzle, passes through a skimmer and is scattered off the sample surface. By varying the nozzle temperature, the beam energy of the incident helium beam can be tuned between 9 and 20 meV. In our setup, the angle between the source arm and the detector arm is fixed at 91.5°. After hitting the sample in the main chamber under ultra-high vacuum (UHV) conditions (*p* < 2 ⋅ 10^–10^ mbar) the beam is detected using a quadrupole mass spectrometer. By rotating the sample, the angle of incidence *ϑ*_*i*_ can be varied.

TaS_2_ crystals were grown by chemical vapor transport using I_2_ as transport agent and the 1T-TaS_2_ phase was obtained by quenching the crystals from ≈1000° C. 1T-TaS_2_ was further characterized by X-ray diffraction, resistivity measurements, and ARPES (Ngankeu et al., 2017). From single crystal X-ray diffraction, some stacking disorder is observed in the bulk, which does, however, not affect HAS or any other surface sensitive method. The crystals, as shown in Figure 1C, are thin and can only be cleaved a limited number of times *in situ* by the scotch tape method. Prior to the measurements a clean surface was prepared by applying scotch tape to the TaS_2_ sample surface and peeling it off in a UHV transfer chamber (Tamtögl et al., 2016).

The TlBiTe_2_ sample was grown in the group of C. Felser (Li et al., 2017; Pei et al., 2022). Due to the stronger bonding between the layers as illustrated in Figure 1B, cleaving requires knocking off an attached post *in situ* in the mentioned transfer chamber. The latter had been attached with epoxy prior to transferring the sample into the transfer chamber. Figure 1E shows an image of the post with the remaining attached crystal after it had been removed from the chamber.

Following cleavage, both samples can be inserted into the scattering chamber. For temperature-dependent measurements, the sample can be cooled down to 115 K via a thermal connection to a liquid nitrogen reservoir and heated using a button heater.

## 3 Results and discussion

Figures 1A, B illustrate the structures of both 1T-TaS_2_ and TlBiTe_2_. As mentioned in the introduction, it is evident from the side view of TlBiTe_2_ that the coupling between the atomic layers is much stronger and less of a vdW type compared to the TMdCs but also compared to bonding between the quintuple layers of the binary TIs. The latter is actually confirmed in our experiments as TlBiTe_2_ is much more difficult to cleave as mentioned above. In the Auger electron spectroscopy (AES) spectrum shown in Figure 1F mostly the main peaks from Tl and Bi are present, the peak at about 45 eV stems most likely from elements in the sample holder. As said above, TlBiTe_2_ is a trigonal crystal but similar to the binary TIs it is more convenient to consider them in the conventional hexagonal notation (red dashed line in Figure 1B) with *c* = 3*c*_0_, i.e. three repeated elemental sequences. These repeatable sequences, consisting of four layers each, are however compared to the quintuple layers in the binary TIs not separated by a vdW gap.

**FIGURE 2:**
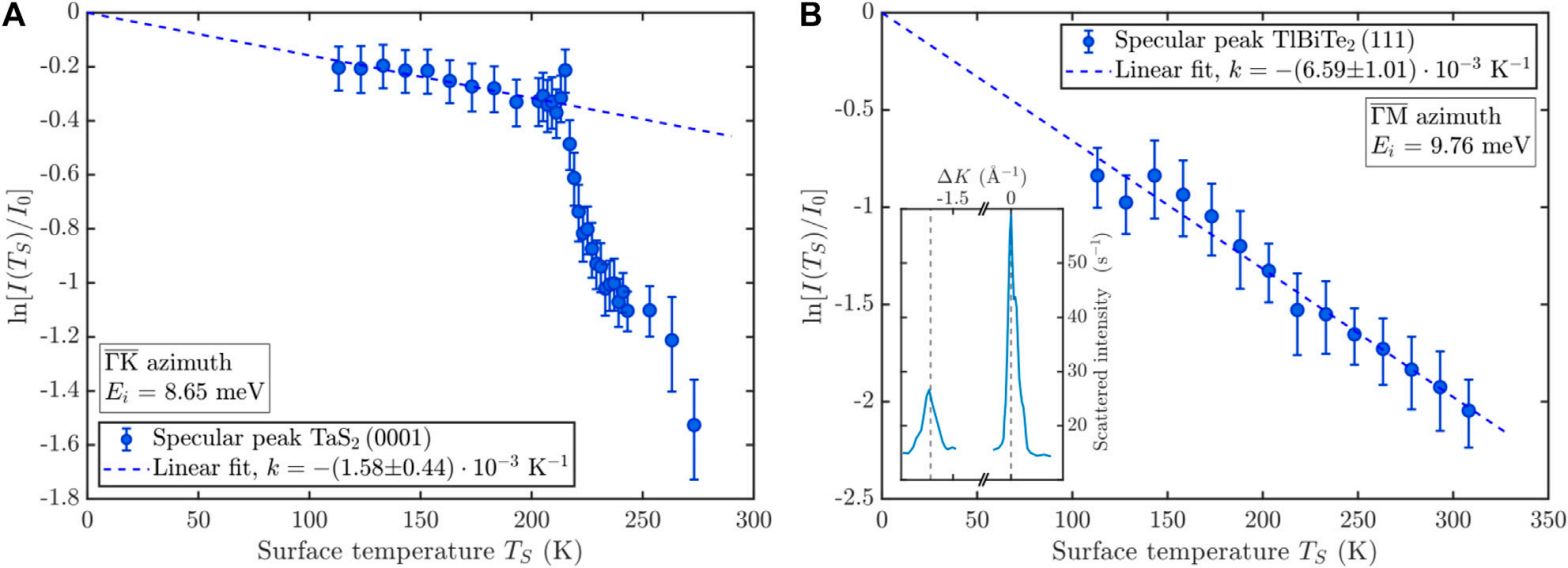
The temperature dependencies of the Debye-Waller exponents of **(A)** 1T-TaS_2_ for the specular peak with the sample aligned along $\bar{\Gamma K}$ and of **(B)** TlBiTe_2_(111) aligned along $\bar{\Gamma M}$ . TlBiTe_2_ exhibits the typical linear behavior over the entire temperature range, whereas, after heating 1T-TaS_2_, the intensity follows a linear decrease within the commensurate CDW phase, followed by a small increase when entering the triclinic CDW phase at around 210 K and an abrupt decrease with a further transition to the incommensurate CDW phase at around 270 K. The inset of **(B)** shows an angular scan of TlBiTe_2_ along $\bar{\Gamma M}$ indicating a rough surface with diffuse scattering.

The surface termination of TlBiSe_2_ has been studied experimentally by Kuroda et al. using scanning tunneling microscopy (STM) and core-level photoelectron spectroscopy (CL-PES) (Kuroda et al., 2013) and by Pielmeier et al. using ARPES, X-ray photoelectron spectroscopy (XPS), STM and atomic force microscopy (AFM) (Pielmeier et al., 2015). Both studies agree that cleaving happens between Tl and Se layers since the bonding strength between these layers is weaker compared to other layers according to *ab initio* calculations (Eremeev et al., 2011). Their STM and CL-PES results showed that after cleavage the surface is terminated by a Se layer with islands of Tl atoms on top of the Se layer covering roughly half of the surface which is further supported by *ab initio* calculations for TlBiSe_2_ and TlBiTe_2_ (Singh et al., 2016). Following the reports which suggest a rough, nonpolar surface model (Kuroda et al., 2013; Singh et al., 2016) it appears likely that the same scenario holds for TlBiTe_2_, i.e., a Te layer covered by Tl islands forms the termination after cleavage. In fact the inset of Figure 2B which shows the first order diffraction and the specular along $\bar{\Gamma M}$ also suggests a rough surface with diffuse scattering due to the small specular which is a measure for the surface order (Farías and Rieder, 1998) and described in more detail in Section 3.2.

**FIGURE 3:**
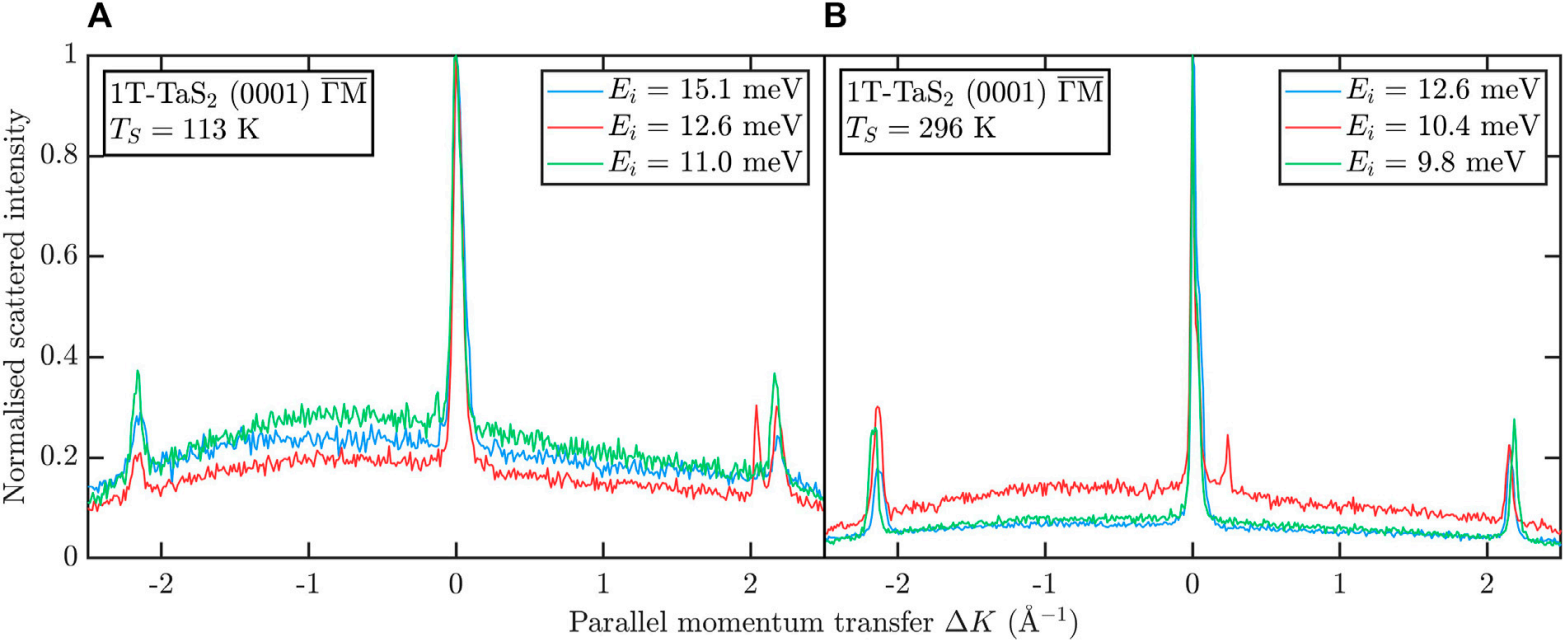
Diffraction scans of TaS_2_ at surface temperatures of **(A)** T_S_ = 113 K and **(B)** T_S_ = 296 K both in $\bar{\Gamma M}$ direction and at various incident beam energies show the corresponding first order diffraction. Even though it is not directly in the scanning direction, a superlattice peak appears in **(A)** only for *E*_*i*_ = 12.6 meV (red curve). The additional peak next to the specular peak in **(B)** for *E*_*i*_ = 10.4 meV is a feature of resonant processes at the surface.

After cleavage of 1T-TaS_2_ on the other hand we can clearly resolve the first order diffraction peaks as illustrated in Figure 3, both at room temperature as well as for the cooled sample. The complex CDW structure of 1T-TaS_2_, as described by Wilson et al. (Wilson et al., 1975; Wilson et al., 2001; Coleman et al., 1992), and more recently by (Yu et al., 2015) has previously been studied with HAS by (Cantini et al., 1980) and Brusdeylins et al. (Brusdeylins et al., 1989; Benedek et al., 2020b). Here we concentrate on a more detailed study and analysis of the nearly commensurate and commensurate CDW phase of 1T-TaS_2_. As described in recent transport measurements, upon cooling 1T-TaS_2_ sequentially enters an incommensurate CDW (I-CDW) phase below 550 K, a nearly commensurate CDW (NC-CDW) phase below 350 K, and finally a commensurate CDW (C-CDW) phase below 180 K (Wang et al., 2020). When comparing the cooling and heating data a hysteretic behavior is typically observed both in resistivity (Wang et al., 2020) as well as in HAS measurements (Benedek et al., 2020b). Moreover, upon heating, 1T-TaS_2_ enters a triclinic CDW (T-CDW) phase at 220 K, followed by the NC-CDW phase at 280 K (Wang et al., 2020). The space modulations of different CDWs form so-called star-of-David clusters which can be clearly seen in the low energy electron diffraction (LEED) pattern in Figure 1D. In the C-CDW phase, the star of David clusters cover the entire lattice, forming a commensurate $\left(\sqrt{13}\times \sqrt{13}\right)$ R13.54°, with the high symmetry $\bar{\Gamma M}$ azimuth of 1T-TaS_2_ highlighted as red dashed arrow in Figure 1D.

In general, weakly coupled layered structures such as TaS_2_ have many polymorphs with different stackings and the contrasting behavior between both investigated samples becomes evident from the Debye-Waller plot in Figure 2. TlBiTe_2_ exhibits only one phase within the covered temperature region and shows the typical linear decrease of the specular intensity. For 1T-TaS_2_ on the other hand, upon heating, the typical linear decrease of the specular intensity within the commensurate CDW phase is followed by a small increase upon entering the triclinic CDW phase at around 210 K and an abrupt decrease with a further transition to the incommensurate CDW phase around 270 K. In the following, we describe how HAS permits to determine the e-ph coupling and compare the obtained values for both systems.

### 3.1 Electron-phonon coupling from temperature dependent atom-scattering measurements

A convenient parameter to characterize the e-ph coupling strength is the mass-enhancement *λ* (Grimvall, 1981). Since the e-ph coupling describes the interaction between the electronic system and the lattice dynamics (phonons), experimental studies at finite temperatures can either concentrate on the electronic or the phononic system. The former is mostly available via ARPES by examining the renormalization of the electron energy dispersion in the vicinity of the Fermi surface as a result of e-ph interactions. The latter can be carried out using HAS, which on the contrary studies the renormalisation of the surface phonon dispersion due to e-ph interactions. As shown in recent works (Tamtögl et al., 2017; Benedek et al., 2018; Benedek et al., 2020c; Manson et al., 2022), the temperature dependence of the Debye-Waller (DW) exponent plotted in Figure 2 permits to extract, for a conducting surface, the mass-enhancement parameter *λ*. The Debye-Waller factor takes into account the thermal attenuation of the elastic helium intensity due to atomic motion. The attenuated intensity *I*(*T*_*S*_) with respect to *I*_0_, the intensity at rest (*T*_*S*_ = 0 K) in the absence of zero-point motion is given as

$$I\left(T_{S}\right)=I_{0}e^{-2W\left(k_{i},k_{f},T_{S}\right)},$$ (1)

with **k**_*i*_ and **k**_*f*_ being the wave vectors before and after scattering, respectively. Since a He beam with energies in the meV region is scattered on a conducting surface by the surface free electron density, the exchange of energy with the phonon gas occurs via the phonon-induced modulation of the surface electron gas, that is, via the e-ph interaction (Benedek et al., 2020c; Tamtögl et al., 2021a; Manson et al., 2022). Therefore, the DW-factor, originating from the integrated action of all phonons weighted by their respective Bose factors, turns out to be directly proportional, under reasonable approximations, to the mass-enhancement factor *λ* (Manson et al., 2022).

The relation between *λ* and the DW-exponent is given by the equations:

$$\begin{array}{c} \lambda =\frac{\pi }{2n_{s}}\alpha ,\;\alpha \equiv \frac{\phi }{A_{c}\;k_{iz}^{2}}\frac{\partial \ln I\left(T_{S}\right)}{k_{B}\;\partial T_{S}}, \end{array}$$ (2)

where *ϕ* is the work function, *A*_*c*_ the unit cell area, *I*(*T*_*S*_) the He-beam specular intensity, *T*_*S*_ the surface temperature, *k*_*iz*_ the normal component of the incident wavevector and *n*_*s*_ the number of conducting layers which contribute to the phonon-induced modulation of the surface charge density. The latter is estimated to be *n*_*s*_ = 2*λ*_TF_/*c*_0_, where *λ*_*TF*_ is the Thomas–Fermi screening length characterizing the surface band-bending region, *c*_0_ the thickness of the cleaved layer, and the factor two considers two metallic sheets per layer.

The advantage of HAS is that it directly provides a surface sensitive measure of the mode-averaged *λ*. On the contrary, with ARPES a dependence of *λ* on the initial-state electron energy has been reported (Kumar et al., 2022) and it remains difficult to distinguish between surface and bulk states which is why often time-resolved methods are used (Sobota et al., 2023). Similarly, Raman measurements provide access to the e-ph interaction of optical modes, but again, the method is rather bulk sensitive (Shojaei et al., 2021).

#### 3.1.1 Electron-phonon coupling of 1T-TaS_2_

As described above, from the slopes of the HAS specular intensity as a function of surface temperature the corresponding e-ph coupling can be determined using Eq. 2. We follow here the transition from the C-CDW phase to the NC-CDW phase more closely via individual small diffraction scans over the specular peak. According to Benedek et al. (2020b) the observed specular intensity slopes in the corresponding phases give *λ*(C-CDW) = (0.61 ± 0.06), *λ*(NC-CDW) = (0.91 ± 0.09), and *λ*(I-CDW) = (0.61 ± 0.10) for the three phases, which neglects however the triclinic phase (T-CDW) upon heating.

Using the slope in Figure 2A obtained in the C-CDW phase for the calculation of *λ* together with *k*_*iz*_ = 2.85 Å^−1^, *ϕ* = 5.2 eV (Shimada et al., 1994), *A*_*c*_ = 9.48 Å^2^ and *n*_*s*_ = 3.28 from *c*_0_ = 6.1 Å and a Thomas–Fermi screening length not exceeding 1 nm (Yu et al., 2015) we obtain:

$$\lambda \left(\text{C-}\text{CDW}\right)=0.59\pm 0.12.$$

The e-ph coupling *λ* in the C-CDW phase is thus consistent with the value obtained by Benedek et al. (2020b), although for *n*_*s*_ = 2 as in the mentioned work we would obtain an even larger value with *λ* = 0.97 ± 0.19.

On the other hand, as the attractive part of the surface potential with *D* = 8.4 meV (see Section 3.4 for an exact determination) is comparable to the kinetic energy of the He atoms (8.65 meV) one needs to correct $k_{iz}^{2}$ to account for the acceleration by the attractive part of the potential on the He atom when approaching the surface turning point [Beeby correction (Farías and Rieder, 1998)]. Therefore, $k_{iz}^{2}$ is replaced by $k_{iz}^{\prime 2}=k_{iz}^{2}+2\;mD/\hbar^{2}$ , where *m* is the He mass and *D* the He-surface potential well depth and considering the Beeby correction, we obtain *λ*(C-CDW) = 0.32 ± 0.06.

In general, low-dimensional materials typically exhibit strong Peierls instabilities and e-ph interactions, and vdW materials such as 1T-TaS_2_ provide an ideal platform to study CDWs and the associated superconductivity (Johannes and Mazin, 2008; Rossnagel, 2011). However, while optical methods provide an effective tool to identify the transition temperature of the CDW phase (Lai et al., 2021), quantitative experimental reports about the mode-averaged *λ* are again quite scarce (Clerc et al., 2006). Compared to the e-ph coupling of TlBiTe_2_ as described below, it is evident that on the 1T-TaS_2_ surface and, in particular in the C-CDW phase a much stronger e-ph coupling *λ* is present. Further HAS studies that follow the temperature dependence of the DW-exponent in other TMdDCs obtain similar values for *λ* with an overview being given in Benedek et al. (2020a).

#### 3.1.2 Electron-phonon coupling of TlBiTe_2_

For TlBiTe_2_ we use Eq. 2 together with *ϕ* = 4.2 eV (National Institute of Standards and Technology, 2018), *A*_*c*_ = 18.2 Å^2^ and *k*_*iz*_ = 3.02 Å^−1^. The Thomas–Fermi screening length *λ*_TF_ could be estimated according to the formula used for TMdCs such as 2H-MoS_2_ with $\lambda_{\text{TF}}={\left(\frac{\hbar^{2}\epsilon_{\text{r}}}{4m^{*}e^{2}}\right)}^{1/2}{\left(\frac{\pi c}{3n_{c}}\right)}^{1/6}$ , yielding with Refs (Lubell and Mazelsky, 1965; Paraskevopoulos, 1985)^1^ *λ*_TF_ ≈ 30 Å. However, since TlBiTe_2_ is a 3D TI, *λ*_TF_ ≈ 60 Å similar to the values found for other TIs such as Bi_2_Se_3_ and Bi_2_Te_2_Se seems to be more appropriate (Benedek et al., 2020c). We further note that such a large *λ*_TF_ compared to the TMdCs is supported by *ab initio* calculations by Eremeev et al. (2011) which found that the conducting Dirac state penetrates deep into the bulk and even for slabs of 23-layer thickness (≈55Å) an almost unsplit Dirac cone was obtained. Thus, with *c*_0_ = 7.89 Å the quadruple layer thickness (Eremeev et al., 2011) and the experimental DW derivative with respect to *T*_*S*_ in Figure 2B, we obtain

λ=0.21±0.03.

The factor 2 for *n*_*s*_ in Eq. 2 is here again needed since *c*_0_ encompasses two distinct metal layers, i.e., Tl and Bi. It should be noted that, unlike in the case of low-index metal surfaces, characterized by a soft-wall repulsive potential and negligible corrugation, here the large electronic corrugation (Ruckhofer et al., 2019) implies again a hard-wall potential with an attractive part comparable to the kinetic energy of the He atoms. With the Beeby correction and *D* = 6.22 meV for the He-surface potential well depth (Tamtögl et al., 2021b) it is found:

λ=0.09±0.01.

While no values for TlBiTe_2_ have been reported, it is interesting to compare the value to the ones found for other TIs. It appears that *λ* found for TlBiTe_2_ is smaller compared to the binary TIs such as Bi_2_Se_3_ while at the same time, considering the Beeby correction, equal to the value found for Bi_2_Te_2_Se (Benedek et al., 2020c) with *λ* = 0.09. As the latter exhibits in analogy to TlBiTe_2_ only a single Dirac cone, it confirms previous reports that an appreciable part of the e–ph interaction is provided by quantum well states as present on the binary TIs. Finally, *λ* seems to be certainly much smaller compared to the e-ph coupling of the archetypal CDW system in 1T-TaS_2_ as described above. In general, a rather small *λ* is also in line with the fact that TlBiTe_2_ does not enter a superconducting phase or if so only at very low temperatures (Benedek et al., 2020b). For example, for early reports about a superconducting phase of bulk TlBiTe_2_ well below 1 K by Hein and Swiggard (1970) it was later argued that it may rather be due to a phase separation of TlTe from BiTe (Popovich et al., 1984). Finally, compared to other topological materials, i.e., in Weyl semimetal systems, recent theoretical reports of the bulk e-ph coupling *λ* vary between values of 0.13 for NbAs and 0.43 for TaAs (Han et al., 2023), where in both cases the major contribution comes from acoustic phonon modes. These results suggest an equal or even more significant *λ* compared to TIs such as TlBiTe_2_. However, experimental approaches in connection with Weyl materials are mainly based on temperature-dependent Raman studies, thus focusing on optical phonon modes and being rather bulk sensitive (Xu et al., 2017; Choe et al., 2021; Osterhoudt et al., 2021; Al-Makeen et al., 2022). In light of these results, surface-sensitive experimental reports about the average e-ph coupling *λ* of Weyl semimetals are not available to the best of our knowledge, preventing us from providing a decent comparison with these.

### 3.2 Helium scattering and diffraction from 1T-TaS_2_ and TlBiTe_2_

Since thermal helium atoms have a wavelength comparable to inter-atomic distances the elastic scattering of a helium beam from a periodic surface gives rise to a diffraction pattern. The distances between the diffraction peaks can then be used to calculate the surface lattice constant and in a second step the in-plane linear thermal expansion coefficient. Figure 3 shows several recorded angular diffraction scans (*ϑ*-scans) obtained by rotating the sample in the scattering plane. Conversion to parallel momentum transfer Δ*K* follows from

$$\Delta K=|\Delta K|=|K_{f}-K_{i}|=|k_{i}|\left(\sin\vartheta_{f}-\sin\vartheta_{i}\right),$$ (3)

with **k**_*i*_ being the incident wave vector and *ϑ*_*i*_ and *ϑ*_*f*_ the incident and final angles with respect to the surface normal, respectively. The scans in Figure 3 were taken along the high symmetry $\bar{\Gamma M}$ orientation keeping the surface at temperatures of *T*_*S*_ = 113 K (left panel) and 296 K (right panel), respectively. The intensity of each specular peak was used for normalization. All scans show clearly a very pronounced specular peak (*ϑ*_*i*_ = *ϑ*_*f*_) and two first order diffraction peaks.

The small side peak next to the specular peak for the scan with *E*_*i*_ = 10.4 meV occurs due to the presence of the atom-surface potential, which will be further described in Section 3.4. In addition to diffraction peaks, the angular scan at *E*_*i*_ = 12.6 meV and *T*_*S*_ = 113 K (red curve in the left panel of Figure 3) shows a very sharp satellite peak next to the (1,0)-diffraction peak at Δ*K* = 2.04 Å. At this temperature, the crystal is in the C-CDW phase, which is characterized by the star-of-David clusters covering the entire lattice forming a commensurate $\left(\sqrt{13}\times \sqrt{13}\right)$ R13.54° superstructure. Interestingly, while we performed diffraction scans at various nozzle temperatures, the satellite peak is only visible for *E*_*i*_ = 12.6 meV and secondly, we are able to resolve the peak even though it should be rotated with respect to the scanning direction as illustrated in Figure 1D. The latter could be due to a slight azimuthal misalignment of the sample, although we note that Brusdeylins et al. (1989) also observed the peaks arising from the corresponding superstructure, not only in the C-CDW phase but also in the NC-CDW phase at *T*_*S*_ = 340 K.

For TlBiTe_2_ on the other hand, due to the difficulty associated with the cleavage and the reported likelihood of a patchy termination, we are only able to resolve the specular and a small diffraction peak on one side as shown in the inset of Figure 2B. In general, the intensity scattered into the specular direction is typically by a factor of 10 higher for 1T-TaS_2_. As described above, this is sufficient for a determination of the e-ph coupling strength of TlBiTe_2_ however, it does not permit us to perform any detailed analysis of the TlBiTe_2_ surface structure and electronic corrugation.

Nevertheless, since a measurement of the angular spread in the specular peak provides an estimate of the surface quality (Farías and Rieder, 1998) and following the approach described in Tamtögl et al. (2017) we can use the measured full-width at half maximum (FWHM) of the specular peak to compare surface order and more specifically the terrace width for both samples.

The peak broadening is proportional to the average domain size, also known as the surface coherence length. As the measured specular width Δ*θ*_*exp*_ is a convolution of the angular broadening of the apparatus Δ*θ*_*app*_ and the broadening form the domain size Δ*θ*_*w*_, it follows: $\Delta \theta_{exp}^{2}=\Delta \theta_{w}^{2}+\Delta \theta_{\mathrm{app}}^{2}$ . The coherence length can then be determined using:

$$l_{c}=\frac{5.54}{\Delta \theta_{w}\;k_{i}\;\cos\vartheta_{f}}$$ (4)

with *k*_*i*_ and *ϑ*_*f*_ as defined beforehand (Farías and Rieder, 1998; Tamtögl et al., 2017).

For 1T-TaS_2_, the measured FWHM is typically about 0.04° in *ϑ*_*i*_-scans, which corresponds to a terrace width of ≈500 Å. For TlBiTe_2_ the FWHM is about 0.05 Å^−1^ in momentum space (inset of Figure 2B) or 0.07° in *ϑ*_*i*_ giving rise to a terrace width of ≈200 Å. While STM studies report a Tl island size of the order of 1 − 2 nm and thus much smaller than our result, observed terraces in STM measurements exhibit also widths of about 20 nm (Kuroda et al., 2013; Pielmeier et al., 2015). As the absence of the Tl islands in room temperature STM measurements was attributed to increased mobility of the Tl atoms (Pielmeier et al., 2015), we note that addressing these details and differences would require additional measurements and a more in-depth study.

In summary, we conclude, that as already anticipated, TlBiTe_2_ is “rougher” and exhibits smaller domain sizes compared to 1T-TaS_2_. However, neither of the surface qualities is comparable to some of the binary TIs, where the angular broadening of the specular peak had been reported to be mainly limited by the angular broadening of the apparatus (Tamtögl et al., 2017; Ruckhofer et al., 2019).

### 3.3 Thermal expansion of 1T-TaS_2_

Its unique surface sensitivity makes He diffraction an ideal method to determine the lattice constant of solely the surface layer without any contribution from the underlying layers. From our angular diffraction scans we can thus calculate the surface lattice constant of 1T-TaS_2_ and in a second step the linear thermal expansion coefficient. Following the two-dimensional Laue condition for a hexagonal lattice, the surface lattice constant can be calculated. For *T*_*S*_ = 113 K, we recorded 14 angular scans in $\bar{\Gamma M}$ direction at different incident beam energies resulting in an average lattice constant of *a* = (3.35 ± 0.03) Å. Similarly, we used eight scans in $\bar{\Gamma M}$ direction for the lattice calculation at *T*_*S*_ = 296 K. From the position of the first order diffraction peaks with respect to the specular peak we thus obtain a surface lattice constant of *a* = (3.37 ± 0.03) Å at room temperature, which is an increase of roughly 0.6%.

Both values are in good agreement with other experimental studies (scanning tunneling microscopy, LEED, X-ray powder diffraction, surface-enhanced Raman spectroscopy (SERS), and theoretical studies using density functional theory) of the 1T-TaS_2_ in-plane lattice parameter (Wilson et al., 1975; Givens and Fredericks, 1977; Sanders et al., 2016; Kratochvilova et al., 2017; Bao et al., 2022).

With these values we can further calculate the in-plane (linear) thermal expansion coefficient (TEC) defined as *α*_‖_ = 1/*a*_0_ ⋅Δ*a*/Δ*T*, where *a*_0_ is the lattice parameter at *T*_*S*_ = 113 K, Δ*a* the difference between the lattice constant values at different temperatures and Δ*T* the temperature difference. We obtain a value of

$$\alpha_{\|}=\left(33\pm 12\right)\cdot 10^{-6}\;K^{-1}.$$

Our value is larger compared to the value that Givens and Fredericks obtained in their X-ray diffraction (XRD) study. As we have only considered two different surface temperatures our result should be treated carefully even though it seems unlikely to be caused by such a large uncertainty and might well be a consequence of the different probing techniques. Table 1 provides a short overview of the thermal expansion coefficients of other transition metal dichalcogenides, which experimental technique had been used, and for which temperature range. Previous studies of TMdCs reported values for the TEC in the range of 5–25 ⋅ 10^–6^ K^−1^, thus, comparable but slightly smaller than our value (Anemone et al., 2022), which may point toward a particularly weak vdW coupling between the layers.

**TABLE 1:** Overview of the in-plane thermal expansion coefficients (TEC) of several TMdCs, how they were obtained and for which temperature range. In the case of a negligible TEC the upper limit is noted.

Material	Method	α_‖_ (10^–6^ K^−1^)	*T*_*S*_ range (K)
MoS_2_ (Anemone et al., 2018; Anemone et al., 2022)	HAS	≤14.0	90–522
MoSe_2_ (El-Mahalawy and Evans, 1976)	XRD	7.2	293–1073
PdTe_2_ (Anemone et al., 2022)	HAS	≤24.8	90–290
PtTe_2_ (Anemone et al., 2020)	HAS	≤5.6	90–550
TaS_2_ (Givens and Fredericks, 1977)	XRD	12.7	138–482
TaS_2_ (This work)	HAS	32.9	113–296
WS_2_ (Zhang et al., 2016)	SERS	10.3	110–300
WSe_2_ (Brixner, 1963; El-Mahalawy and Evans, 1976)	XRD	6.8–11.1	25–600

### 3.4 Atom-surface interaction and surface electronic corrugation of 1T-TaS_2_

As every polarisable object in nature is subjected to the vdW force a fundamental understanding of the force is crucial for any quantitative description and theoretical treatment of molecular adsorption or surface reaction processes and is also of paramount importance for a better design and control of nanoscale devices. Beyond the applications of weak interactions in devices and materials, there are several experimental techniques analyzing nano-structured materials or explicitly using effects occurring from these weak interactions including atomic force microscopy and matter-wave scattering experiments which are probing dispersion forces (Fiedler et al., 2023).

However, in the case of topological nontrivial materials, HAS is the only experimental technique used to date to provide experimental information about the vdW interaction with a topological insulator (Tamtögl et al., 2021b). The latter stresses the need for experimental measurements even more so as, e.g., it is expected that peculiar effects such as the topological magnetoelectric effect (Dziom et al., 2017) cause an unconventional contribution to the vdW potential (Martín-Ruiz and Urrutia, 2018). Before we illustrate the experimental determination of the atom-surface vdW potential of 1T-TaS_2_ we will first describe HAS measurements of the step-height distribution in the following.

**FIGURE 4:**
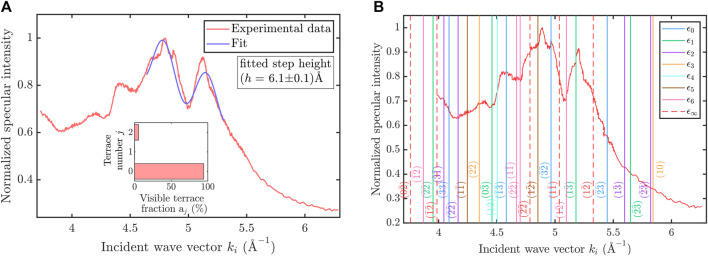
**(A)** Periodic oscillations in the measured specular intensity *versus* incident wave vector *k*_*i*_ provide a measure for the step height distribution of the 1T-TaS_2_(0001) sample. Following an analysis upon fitting Eq. 6 (blue curve) to the experimental data points (red) we obtain a step height *h* = (6.1±0.1) Å for the measurement performed along the $\bar{\Gamma M}$ azimuth while the crystal was kept at a temperature of 296 K **(B)** The position of selective adsorption resonances following an analysis of the same drift spectrum as in **(A)** allows to determinate the laterally averaged atom-surface interaction potential. The vertical colored lines correspond to the different bound-state energies *ϵ*_*n*_ with the dashed red lines illustrating the threshold energy and the corresponding *G*-vector as a label.

The surfaces of layered materials such as 1T-TaS_2_ are not perfectly flat, rather they are characterized by steps and parallel terraces, e.g., due to defects and the sample preparation process. The step heights and distributions of such periodically modulated surfaces can be calculated and resolved from interference effects in HAS (Farías and Rieder, 1998; Mayrhofer-Reinhartshuber et al., 2013a). As a result of the combination of the He beam being scattered from different terraces, constructive and destructive interference occurs. In a so-called drift measurement, the specular intensity is monitored while the kinetic energy of the incident He beam is changed. In the experiment this can be achieved by changing the nozzle temperature. By modulating the kinetic energy, the phase shift Δ*ϕ* for the He beam emerging from different terraces is varied. For the specific case of *ϑ*_*i*_ = *ϑ*_*f*_ (specular peak) the phase difference of two adjacent terrace levels is given by (Mayrhofer-Reinhartshuber et al., 2013a)

$$\varphi \left(k_{i}\right)=2hk_{i}\cos\vartheta_{i}=h\Delta k_{z},$$ (5)

where *h* is step height and Δ*k*_*z*_ is the change of the wave vector component perpendicular to the surface. For constructive interference, the condition of in-phase scattering Δ*ϕ* = 2*πn* with an integer value of *n* must be fulfilled, whereas a half-integer value *n* leads to destructive interference due to anti-phase scattering. As a result, maxima and minima can be observed in the drift spectrum Figure 4A, where the normalized specular intensity is plotted against the incident wave vector *k*_*i*_. The measured specular intensity as a function of incident wave vector *k*_*i*_ upon varying the nozzle temperature is plotted for the sample aligned along $\bar{\Gamma M}$ and the crystal being held at room temperature.

As the incident wave vector *k*_*i*_ is varied by changing the nozzle temperature *T*_*N*_, the He beam intensity scales via $I\propto 1/\sqrt{T_{N}}$ and the signal has to be corrected for this factor which has been done in Figure 4A. In addition, the intensity decreases also with increasing *k*_*i*_ according to the Debye-Waller attenuation (Ruckhofer et al., 2019). Such a drift spectrum can now be used to determine the step height of the current 1T-TaS_2_ sample.

Following a simple theoretical model to describe the periodic intensity modulation which assumes a coherent overlap of plane waves, emerging from different terrace levels, the specular intensity can be calculated with (Tölkes et al., 1997; Mayrhofer-Reinhartshuber et al., 2013a)

$$I\left(k_{i}\right)=I_{0}\;e^{-2W}\;{\left|\overset{\infty }{\underset{j=0}{\sum }}a_{j}e^{-ij\varphi \left(k_{i}\right)}\right|}^{2}.$$ (6)

In Equation 6, *I*_0_ is the intensity for a flat surface without steps, *φ*(*k*_*i*_) the phase shift (see Eq. 5) and *a*_*j*_ the visible fraction of the terrace level *j*. Eq. 6 has been fitted to the measured drift spectrum in the wave vector range 4.54 Å^−1^ < *k*_*i*_ < 5.41 Å^−1^ and the best fit result is shown in Figure 4 as a blue line with the inset indicating the distribution of the visible terrace fractions. We obtain a surface step height of *h* = (6.1 ± 0.1) Å, which is in excellent agreement with the distance between two adjacent TaS_2_ layers (Bovet et al., 2003; Li et al., 2019; Kahraman et al., 2020).

As mentioned above, HAS provides direct experimental information about the vdW interaction between an atom and the surface and thus invaluable details about London dispersion forces of vdW layered materials. As a first step to determine the three-dimensional corrugated atom-surface interaction potential of 1T-TaS_2_, we follow a further analysis of the drift spectrum. The simple model for interference effects from steps (see Eq. 6) neglects the occurrence of selective adsorption resonances (SARs). These phenomena occur when an incoming helium atom is bound temporarily on the surface due to the attraction of the He-surface interaction potential. A SAR follows from the kinetic condition that the energy of the incident energy *E*_*i*_ equals the kinetic energy of the atom on the surface plus the binding energy *ϵ*_*n*_ of the potential:

$$E_{i}=\frac{\hbar^{2}k_{i}^{2}}{2m}=\frac{\hbar^{2}}{2m}{\left(K_{i}+G\right)}^{2}+\epsilon_{n}\left(K_{i},G\right).$$ (7)

Such adsorption processes give rise to peaks and dips in the drift spectrum (Tamtögl et al., 2021b). Therefore, these peaks and dips can be used to obtain the bound state energies of the potential. As a first step we concentrate on the laterally averaged atom-surface potential. Each peak/dip at a specific incident wave vector *k*_*i*_ can be associated with a certain bound state energy *ϵ*_*n*_ and reciprocal lattice vector *G* pair which fulfills the condition given in Eq. (7). In the so-called free atom approximation, *ϵ*_*n*_ (**K**_*i*_, **G**) is independent of **K**_*i*_ and **G** (Ruckhofer et al., 2019; Tamtögl et al., 2021b) and thus Figure 4B provides a direct measure of the bound state energies *ϵ*_*n*_ of the laterally averaged potential.

As a next step, we will then model the three-dimensional He-surface interaction potential, whereupon we use the corrugated Morse potential (CMP) due to its algebraic simplicity (Tamtögl et al., 2021b). It consists of two exponentials considering the repulsive and the attractive part of the potential as a function of the lateral position **R** on the surface and the distance *z* from the surface in the following form:

$$V\left(R,z\right)=D\left[\frac{1}{\nu_{0}}e^{-2\kappa \left[z-\xi \left(R\right)\right]}-2e^{-\kappa z}\right].$$ (8)

Here, *κ* is the stiffness and *D* the well depth of the potential, *ξ*(**R**) the corrugation function, which reflects the periodicity of the crystal surface, and *ν*_0_ is the surface average over *e* ^2*κξ*(**R**)^. The laterally averaged surface potential (i.e., without corrugation in the exponential) of Eq. 8 can be described analytically and the bound state energies are given by

$$\epsilon_{n}=-D+\hbar \omega \left(n+\frac{1}{2}\right)\left(1-\frac{n+\frac{1}{2}}{2\gamma }\right)$$ (9)

with *n* being a positive integer, $\omega =\kappa \sqrt{2D/m}$ the Debye-frequency and *γ* = 2*D*/*ℏω*. By fitting Eq. 9 to the *ϵ*_*n*_-values obtained from the SAR analysis of the drift spectrum we can thus obtain the laterally averaged potential with the well depth and stiffness:

$$D=\left(8.40\pm 0.15\right)\;meV\;and\;\kappa =\left(0.45\pm 0.02\right)\;{Å}^{-1}.$$

**FIGURE 5:**
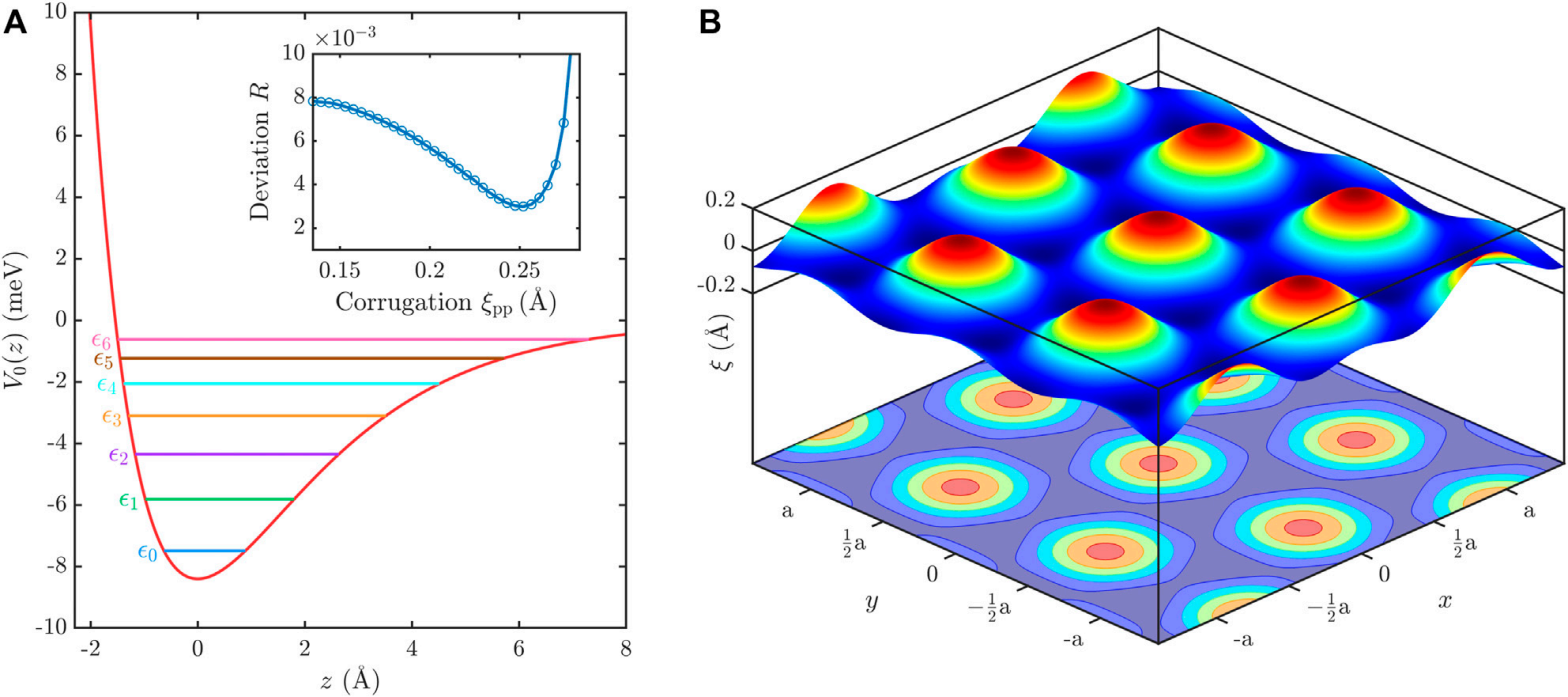
**(A)** Laterally averaged Morse potential for He-TaS_2_ with *D* = 8.40 meV and *κ* =0.45 Å^−1^ with the first 7 bound state energies *ϵ*_*n*_. The inset shows the deviation *R* (Eq. 11) between CC-calculations and the experimental intensities upon varying the corrugation *ξ*_pp_ with a clear minimum at *ξ*_pp_ = 0.25 Å. **(B)** Shows a combined three-dimensional and contour plot of the obtained surface electronic corrugation *ξ*(*x*, *y*) as part of the three-dimensional corrugated Morse potential.

The resulting bound state energies and their uncertainties are displayed in Table 2 while in Figure 5A a plot of the laterally averaged potential is shown. The positions of the corresponding SARs in the drift spectrum are displayed as vertical lines in Figure 4B, where each color belongs to one specific bound state energy *ϵ*_*n*_ and the red dashed lines illustrate the threshold energies. The labels next to the lines denote the corresponding reciprocal lattice vector *G*. For completeness, it should be mentioned that two more analytical bound state energies *ϵ*_7_ and *ϵ*_8_ exist, but they are quite close to zero, that is, to the threshold condition and therefore not displayed in Figure 4B and Table 2. In Figure 5A, the horizontal colored lines represent the first seven analytically calculated bound state energies. We note that SAR measurements are typically obtained with a cooled sample since with increasing temperature inelastic channels give rise to a broadening of the linewidth and may also cause changes from maxima to minima and *vice versa* (Tamtögl et al., 2018; Tamtögl et al., 2021b). Here we use measurements taken at room temperature as the position of the resonances becomes clearer at that temperature. It may be a consequence of the different phases of the cooled sample and the increased likelihood of rest gas adsorption for the latter.

**TABLE 2:** Experimentally determined bound state energies *ϵ*_*n*_ for 1T-TaS_2_ and corresponding uncertainties upon fitting a laterally averaged Morse potential (Eq. 9) with *D* = 8.40 meV and *κ* = 0.45 Å^−1^.

Bound state	*ϵ*_*n*_ (meV)	Δ*ϵ*_*n*_ meV
*ϵ*_0_	7.55	0.15
*ϵ*_1_	5.86	0.13
*ϵ*_2_	4.38	0.11
*ϵ*_3_	3.11	0.08
*ϵ*_4_	2.06	0.06
*ϵ*_5_	1.23	0.03
*ϵ*_6_	0.61	0.01

Our value of *D* is slightly smaller than the value *D* = 8.7 meV from Brusdeylins *et al.* whose value is based on intensity calculations (Brusdeylins et al., 1989) following the hard corrugated wall model together with the Beeby correction and thus does not directly consider scattering from a soft potential resulting in less accuracy compared to the elastic close-coupling method used in the previous works (Sanz and Miret-Artés, 2007; Mayrhofer-Reinhartshuber et al., 2013b). In general compared to other semiconductors, metals, and semimetals, 1T-TaS_2_ exhibits one of the “deepest” atom-surface interaction potentials, only surpassed by the graphene/graphite potential (Benedek and Toennies, 2018). At the same time, the stiffness *κ* is quite close to the value found for the topological insulator Bi_2_Se_3_ (Ruckhofer et al., 2019; Tamtögl et al., 2021b). With the outermost layer for Bi_2_Se_3_ being Se, located in the same column of the periodic table just below sulphur, it seems reasonable that the He−TaS_2_ potential exhibits a similar stiffness and supports a similar number of bound states.

Following the results of the laterally averaged atom-surface interaction potential we continue with the determination of the three-dimensional atom-surface interaction potential and the corresponding surface electronic corrugation (Tamtögl et al., 2021b; Allison et al., 2022). Following the CMP (Eq. 8), the corrugation *ξ*(**R**) for a hexagonal surface such as 1T-TaS_2_, can be described by a two-parameter Fourier ansatz,

$$\xi \left(R\right)=\xi \left(x,y\right)=\xi_{0}\left[\cos\left[\frac{2\pi }{a}\left(x-\frac{y}{\sqrt{3}}\right)\right]+\cos\left(\left[\frac{2\pi }{a}\left(x+\frac{y}{\sqrt{3}}\right)\right]\right)+\cos\left(\frac{2\pi }{a}\frac{2y}{\sqrt{3}}\right)\right]+h.o.,$$ (10)

where *x* and *y* depict the coordinates in the surface plane and *ξ*_0_ determines the corrugation amplitude. The ansatz above is based on the sixfold symmetry of the topmost surface layer. As the He beam provides an average of the long-range order, we omit the star of David clusters in this description. The corrugation is then typically expressed in terms of the peak-to-peak value *ξ*_pp_ = max(*ξ*) − min(*ξ*). With the CMP (Eq. 8) we are able to calculate the diffraction intensities and compare those with the experimentally measured ones to obtain a value for the peak-to-peak corrugation *ξ*_pp_.

A theoretical framework to describe the elastic scattering of He atoms from surfaces is the close-coupling (CC) formalism, which is more exact than the hard-wall approximation, but still computationally manageable (Sanz and Miret-Artés, 2007; Allison et al., 2022). The starting point here is the time-independent Schrödinger equation together with the CMP, which contains the Fourier series expansion (Eq. 10) in the exponentials, yielding a set of coupled differential equations for the diffracted waves. These equations are solved for using the CC algorithm, taking into account all open channels and 110 closed channels. The *z* − boundaries of the integration were set to [−6, 18]. A detailed theoretical treatment and the corresponding equations can be found in several references (Miret-Artés, 1995; Sanz and Miret-Artés, 2007; Mayrhofer-Reinhartshuber et al., 2013b; Kraus et al., 2015), while an implementation of the CC algorithm is available from https://repository.tugraz.at/records/cd0y0-xa478 under the GNU General Public License v3.0.

By comparing the elastic diffraction intensities calculated with the close-coupling algorithm we are able to determine the corrugation of the sample surface (Ruckhofer et al., 2019; Schmutzler et al., 2022). For the measured diffraction intensities we used the peak areas instead of the peak height to account for broadening due to the energy spread of the He atoms, angular resolution of the apparatus, and size effects of the crystal surface (Ruckhofer et al., 2019). For the CC calculations, the intensities have been corrected by the Debye-Waller factor, and the values for *D* and *κ* from above were kept constant, while the corrugation amplitude *ξ*_0_ (see Eq. 10) was varied between 0.05 and 0.90 Å^−1^. The best corrugation value *ξ*_0_ is the one that minimises the deviation *R* between simulated and experimentally obtained diffraction intensities $I_{G}^{\text{sim}}$ and $I_{G}^{\exp}$ given by

$$R=\frac{1}{N}\sqrt{\underset{G}{\sum }{\left(I_{G}^{\exp}-I_{G}^{\text{sim}}\right)}^{2}},$$ (11)

with *N* the number of experimentally measured diffraction scans. As for the calculation of the lattice constants we used in total 22 scans at various incident energies for the optimization process of Eq. 11 to find a global minimum of *R* as a function of *ξ*_0_. It results in a surface electronic peak-to-peak corrugation (see inset of Figure 5A) of

$$\xi_{\text{pp}}=0.25\;Å,$$

which corresponds to 7.5% of the surface lattice constant *a*. Figure 5B shows a plot of the best-fit surface electronic corrugation according to Equation 10.

So far, there barely exist any other HAS studies of other TMdCs that determine the electronic corrugation. Anemone et al. studied the 1T-PtTe_2_ surface and provided a rough estimation of the maximum corrugation amplitude with 0.33 Å (Anemone et al., 2020). It should be mentioned that their estimation is based on the HCW model, which makes a direct comparison difficult. Similarly, various low-index metal surfaces have been studied in atomic beam scattering experiments and revealed a significantly smaller corrugation (Benedek and Toennies, 2018). On the other hand, in comparison to the binary topological insulators and single-element semimetals, which have been studied recently with HAS (Tamtögl et al., 2021b) and analyzed in a similar manner, TaS_2_ exhibits a similar surface electronic corrugation.

## 4 Summary and conclusion

The increasing interest in vdW 2D materials makes 1T-TaS_2_ probably one of the best-studied TMdCs and, in fact, 1T-TaS_2_ has already been investigated by HAS over 40 years ago. However, these studies were concentrated on a few limited aspects of the system, and theoretical descriptions at that time relied on simplistic models. In contrast, we have reported a detailed helium atom scattering study of the archetypal charge-density wave system and transition metal dichalcogenide 1T-TaS_2_. The electron-phonon coupling has been compared with measurements of the 3D topological insulator TlBiTe_2_. The electron-phonon coupling *λ* in the C-CDW phase of 1T-TaS_2_ has been determined with *λ* = 0.59 ± 0.12 for 1T-TaS_2_ and is thus much larger than the value for TlBiTe_2_ (*λ* = 0.09 ± 0.01) which is similar to other semimetal and topological insulator surfaces. In fact, since *λ* of TlBiTe_2_ is so close to *λ* found for Bi_2_Te_2_Se we conclude that the existence of the Dirac cone alone, as found for both TIs, does not give rise to a significant e-ph coupling.

The large *λ* of 1T-TaS_2_ on the other hand, may be a consequence both of the CDW phase as well as the weak bonding between the individual dichalcogenide layers. The much stronger inter-layer bonding of TlBiTe_2_ on the other hand, is reflected in the difficulty to cleave the crystals resulting in a very rough surface with a domain size of about 20 nm and overall comparably small scattering intensities. It would also be interesting to establish whether other TMdCs with a vdW layered structure such as 1T-TaS_2_ but different bonding between the dichalcogenide layers exhibit a different *λ* in accordance to the dependence of *λ* on the substrate bonding for metal-supported graphene where *λ* varies between 0.06 and 0.22 (Benedek et al., 2021).

By means of elastic scattering, the structural properties of 1T-TaS_2_ have been studied and a linear in-plane thermal expansion coefficient with *α*_‖_ = (33 ± 12) ⋅ 10^–6^ K^−1^ has been determined, which is larger than the value from X-ray diffraction and comparable to other TMdCs (Givens and Fredericks, 1977; Anemone et al., 2022). Interference and resonance effects which can be observed upon varying the He beam energy provide further information allowing us to determine the step-height between parallel terraces with *h* = (6.1 ± 0.1) Å in accordance with the spacing between the dichalcogenide layers. Based on the mentioned interference effects we are able to determine the three-dimensional atom-surface interaction potential of 1T-TaS_2_. Using a corrugated Morse potential, the laterally averaged potential is best described by a well depth *D* = (8.40 ± 0.15) meV and a stiffness *κ* = (0.45 ± 0.02) Å^−1^. Hence 1T-TaS_2_ exhibits one of the “deepest” atom-surface interaction potentials compared to other semiconductors/(semi)metals, only surpassed by graphene/graphite which may again be an indication of the “pure” vdW nature of the interlayer bonding. The full three-dimensional potential follows then from a comparison of the diffraction intensities with quantum-mechanical scattering calculations. The optimal agreement is achieved for a surface electronic corrugation with a peak-to-peak value of *ξ*_pp_ = 0.25 Å. While the value is larger than for low-index metal surfaces it is comparable to other semiconductor/semimetal surfaces.

## Data Availability

The raw data supporting the conclusions of this article will be made available by the authors, without undue reservation.

## Footnotes

1
*m* ^⋆^ = 0.086 *m*_*e*_, *ϵ*_*r*_ = 56.15 and *n*_*c*_ = 2·10^19^ cm^−3^
